# Titanium Increases the Antioxidant Activity and Macronutrient Concentration in Tomato Seedlings Exposed to Salinity in Hydroponics

**DOI:** 10.3390/plants11081036

**Published:** 2022-04-11

**Authors:** Víctor Hugo Carbajal-Vázquez, Fernando Carlos Gómez-Merino, Ernesto Gabriel Alcántar-González, Prometeo Sánchez-García, Libia Iris Trejo-Téllez

**Affiliations:** Laboratory of Plant Nutrition, College of Postgraduates in Agricultural Sciences Campus Montecillo, Texcoco 56230, Mexico; carbajal.victor@colpos.mx (V.H.C.-V.); fernandg@colpos.mx (F.C.G.-M.); alcantar@colpos.mx (E.G.A.-G.); promet@colpos.mx (P.S.-G.)

**Keywords:** biostimulation, inorganic biostimulants, beneficial elements, saline stress, essential nutrients, *Solanum lycopersicum*

## Abstract

Global climate change affects agriculture and tends to aggravate the effect of various environmental stress factors including soil salinity. Beneficial elements such as titanium (Ti) may improve the performance of plants facing restrictive environments such as saline soils. This research work evaluated the individual effect of sodium chloride (0, 50, and 100 mM NaCl) in solution, that of leaf-applied Ti (0, 500, and 1000 mg L^−1^ Ti), and their interactions on physiological, biochemical, and nutritional variables of tomato (*Solanum lycopersicum* L.) seedlings cv. Rio Grande in a factorial design in greenhouse hydroponics. NaCl reduced seedling height, stem diameter, leaf area, SPAD units, and sugar and K concentrations, and increased antioxidant activity in stems and roots, photosynthetic pigments, sugars. Titanium increased the N, P, K, Ca, Mg, and Ti concentrations in leaves, but the concentration of total sugars in leaves was reduced when applying 500 mg Ti L^−1^. Under moderate salinity conditions (50 mM NaCl) the application of Ti increased the antioxidant activity in roots, while, at all salinity levels tested, Ti increased the concentrations of macro-nutrients and Ti in leaves. Titanium is concluded to have a positive effect on the antioxidant activity and nutrition of seedlings under saline stress conditions.

## 1. Introduction

According to FAO [1] more than 424 million hectares of topsoil (0–30 cm) and 833 million hectares of subsoil (30–100 cm) are salt-affected. Furthermore, approximately 20% of agricultural soils have excessive salt contents [2]. Salinity as a stress factor decreases the osmotic potential of the soil, which severely affects water absorption by plants and, therefore, cell turgor, hindering plant growth and yield, with great losses to the agricultural industry [3,4,5]. Due to weathering processes and the use of wastewater in agricultural irrigation, different concentrations and types of salts can be found in soils, rivers, lakes, and groundwater layers. Sodium chloride (NaCl), sodium sulfate (Na_2_SO_4_), and sodium carbonate monohydrate (NaHCO_3_) are highly soluble salts and the most common in cultivated soils [6].

The high accumulation of the Na^+^, Cl^−^, and SO_4_^2−^ ions at the cellular level in plants induces nutritional and osmotic imbalances, as well as the overaccumulation of reactive oxygen species, which as a whole negatively affects cell metabolism and function. Indeed, cell walls can collapse and cause cell death as a consequence of excessive salt [3]. On the other hand, plants under this type of stress can develop homeostatic control of water and ions, tolerance to Na^+^, and synthesis of low-molecular-weight organic solutes in the cytoplasm (i.e., soluble sugars, organic acids, amino acids, and their methylated derivatives), all this to decrease the cellular osmotic potential and maintain the level of turgor [7,8].

Attempts to reclaim arable lands affected by salinity and maintain a productive agricultural system have proved to be expensive and thus unaffordable, with only temporary success, so the introduction of novel agricultural practices such as plant biostimulation may represent a creditable alternative to cope with salt stress [9,10], especially in horticultural crops such as tomato (*Solanum lycopersicum* L.).

Tomato is the second most important vegetable produced worldwide, just after potato. There is a great diversity of tomato genotypes within this species, which can be cultivated in practically every country of the world, in fields, greenhouses and net houses. Given its versatility, tomato can be consumed either fresh or processed into many different products [11]. In both cases, world production and consumption has exhibited a constant growth rate over the last three decades. Importantly, tomatoes represent a major source of bioactive compounds including lycopene, vitamin C, β-carotene, phenolic compounds, and tocopherol, as well as minerals. Many of these compounds are powerful antioxidants with anti-inflammatory properties that also have the ability to modulate the immune system [12], and the concentrations of such compounds in fruit tissues can be increased under certain salt stress conditions [13,14]. Tomato has been classified as moderately sensitive to salinity and most cultivars currently available on the market may exhibit yield reduction when exposed to growth media with electrical conductivity values above 2.5 dS m^−1^ [15], but the application of certain biostimulants has proven to trigger efficient tolerance mechanisms to cope with salinity [9,16]. Importantly, tomato production worldwide is of high economic value. Worldwide production of fresh and processed tomatoes was 186.8 billion metric tons in 2020, with China being the world’s largest producer, followed by India, Turkey, the United States and Egypt, which together supply 70% of global tomato demand [17]. Since 2015, Mexico has been the leading tomato exporter, reaching 3.7 billion pounds in 2020, which accounts for 90.7% of US total imports of tomatoes [18]. In the last decade, the mean annual growth rate of tomato exports in Mexico has been 5.8% [19], which can still grow with the use of novel technologies such asthe application of biostimulants.

Inorganic biostimulants such as beneficial elements have gained momentum in the development of measures to mitigate abiotic stress factors in agriculture. Titanium (Ti) is a beneficial element found in most rocks, sediments, and sands. It is the ninth most abundant element in the Earth’s crust and the second of the transition metals. Ti has a great affinity for oxygen, thanks to which it can be found naturally as titanium dioxide (TiO_2_) in three forms: anatase (tetragonal form), rutile (tetragonal form), and brookite (orthorhombic form), as well as in the form of ilmenite (FeTiO_3_). These forms of Ti in the soil are insoluble, and therefore not available to plants [20,21,22].

Currently, anatase is one of the most used sources to extract Ti, and China has large deposits of this mineral, which have not been fully exploited. In 2012, the world production capacity of TiO_2_ pigments increased to 6.5 million Mg. By 2016, the United States alone imported 247,000 Mg Ti, representing a 12% increase over the previous year; its main supplier countries were Canada (31%) and China (28%). The extracted Ti is used in alloys (aeronautics, aerospace, jewelry, and medicine), paper, paints, pigments, plastics, and porcelain, among other products. It is estimated that by the year 2025, the global demand for TiO_2_ will increase by 4.1% per year, that is, 8.82 million Mg year^−1^ [22,23].

Nowadays, another form of Ti exists in the environment, TiO_2_ nanoparticles (TiO_2_NPs). Global TiO_2_NPs production is estimated at 88,000 Mg year^−1^; they are used in cosmetics, and as sweeteners and flavor enhancers in foods. This type of material reaches crops mainly through wastewater and wastewater sludge, in addition to air, which causes high availability of this element for plants. However, the mechanisms of access, absorption, and transport of Ti in higher plants are still unknown. In Europe, it has been estimated that TiO_2_NPs residues can reach up to 0.13 mg kg^−1^ soil year^−1^, and in soils irrigated with wastewater, they can reach up to 1200 mg kg^−1^ year^−1^ [21,24].

Several studies classify Ti as a beneficial element. In mung beans (*Vigna radiata* L.), Ti improves root and stem length [25]. In soybean (*Glycine max* L.), Ti increases stem height and biomass [26]. In various legumes such as faba bean (*Vicia faba* L.), the application of Ti increases the concentrations of sugars, amino acids, chlorophyll, and proline [27], and in cucumber (*Cucumis sativus* L.), it increases the concentrations of P and K [28]. The objective of this study was to evaluate the main effect of leaf-applied titanium (at concentrations of 0, 500, and 1000 mg Ti L^−1^) and NaCl (at doses of 0, 50, and 100 mM in the nutrient solution), and their interactions, on some physiological, biochemical, and nutritional variables of tomato seedlings, in order to shed light on the role of Ti in tomato exposed to salt stress.

## 2. Results

The results are presented as they were approached during the statistical analysis. First, we analyze the effects of NaCl as a single factor, followed by Ti as the second single factor, and finally, we analyze the interactions between both factors for each variable studied. 

### 2.1. Seedling Height, Stem Diameter, and Leaf Area

The evaluated NaCl doses (50 and 100 mM) decreased the growth variables from 10 days after treatments (DAT). The applied Ti doses (500 and 1000 mg L^−1^) had no significant effects on the aforementioned variables (Table 1 and Table 2).

Regarding the interactions, treatments with 100 mM NaCl with both 500 and 1000 mg Ti L^−1^ caused growth inhibition of seedlings by 21 and 26% at 10 DAT; by 34 and 38% at 20 DAT, and by 43 and 49% at 30 DAT, respectively, in all cases compared to the control without NaCl and without Ti. Stem diameter decreased by 16.3% at 20 DAT only with the treatment consisting of 100 mM NaCl and 1000 mg Ti L^−1^, compared to the treatment without NaCl and without Ti (Table 1).

Treatments with 50 mM NaCl with both 500 and 1000 mg Ti L^−1^ reduced leaf area by 52 and 51%, compared to the treatment without salinity and without Ti. Similarly, treatments with 100 mM NaCl with both 500 and 1000 mg Ti L^−1^ reduced leaf area by 65 and 76%, respectively, compared to the control (Table 2).

### 2.2. SPAD Units and Photosynthetic Pigments

In this study, the dose with 100 mM NaCl significantly reduced the SPAD units by 12% at 20 DAT compared to the control. At 30 DAT, the 50 and 100 mM NaCl doses reduced the value of the SPAD units by 13 and 34%, respectively, compared to the control. No significant differences were observed in the SPAD readings from the addition of Ti. On the other hand, the 100 mM NaCl treatment with both 500 and 1000 mg Ti L^−1^ decreased the SPAD units by an average of 33% compared to the treatment without salinity and without Ti (Table 2).

In leaf pigments, no significant differences were observed in the main effects of NaCl and Ti. In the total chlorophyll variable, the treatment with 100 mM NaCl and 1000 mg Ti L^−1^ showed a reduction in the concentration of total chlorophyll by 42%, compared to the treatment without salinity and with 500 mg Ti L^−1^; in this case, no beneficial effect of Ti was observed (Table 3).

### 2.3. Total Sugars in Leaves, Stems, and Roots of Tomato Seedlings

The concentration of total sugars in the leaves of seedlings treated with 50 mM NaCl was significantly lower than that of the control, by 26%, while those treated with 100 mM NaCl were not statistically different from the control. In stems, the highest sugar concentration was observed with 50 mM NaCl, which was 30% higher than the control and 34% higher than the 100 mM NaCl treatment. In roots, the concentration of sugars in seedlings treated with 100 mM NaCl was 31% lower than the control (Table 4).

The lowest concentration of sugars in leaves was observed in seedlings treated with 500 mg Ti L^−1^, 26% lower than the control. In stems and roots, the effect of Ti on sugar concentration was not significant. 

Regarding the interactions of factors in leaf tissues, the treatments without salinity both with the dose of 500 and 1000 mg Ti L^−1^ were not statistically different from the treatment without salinity and without Ti. However, there was a statistical difference between both doses (500 and 1000 mg Ti L^−1^). The higher concentration was obtained with the 1000 mg Ti L^−1^ treatment, followed by the treatment with 500 mg Ti L^−1^, that is, the higher dose improved the sugar concentration by 48%, compared to the lower dose. In the treatments with 50 mM NaCl both with 500 and with 1000 mg Ti L^−1^, neither showed significant differences with respect to the treatment with 50 mM NaCl and without Ti. The 50 mM NaCl treatment with 500 mg Ti L^−1^ was statistically different, 41% lower, when compared to the treatment without salinity and without Ti. On the other hand, with the 100 mM NaCl treatment, the treatments with 500 and 1000 mg Ti L^−1^ were not statistically different from the treatment with 100 mM NaCl and without Ti. Nonetheless, the treatment with 100 mM NaCl and 1000 mg Ti L^−1^ was 62% higher when compared to the treatment with 100 mM NaCl and 500 mg Ti L^−1^ (Table 4).

In stems, the treatment without NaCl and with 1000 mg Ti L^−1^ increased the concentration of sugars by 62%, compared to the mean observed in plants exposed to 500 mg Ti L^−1^ in the absence of salinity. Nonetheless, both treatments were statistically similar to the control without salinity and without Ti. The highest concentration of sugars in stems was observed with the 50 mM NaCl treatment without Ti, being statistically superior to the treatment without salinity and without Ti. The treatments with 50 mM NaCl with both 500 and 1000 mg Ti L^−1^ significantly decreased the sugar concentration, by 34 and 46%, respectively, when compared to the treatment with 50 mM NaCl and without Ti. The lowest sugar concentration was observed in the treatment with 100 mM NaCl and with 1000 mg Ti L^−1^; this was 40% lower than the treatment without salinity and without Ti (Table 4).

In roots, NaCl reduced the sugar concentration, while the main effect of Ti and the interaction of factors were not significant (Table 4).

### 2.4. Total Antioxidant Activity

In this study, the antioxidant activity in leaves and stems was only influenced by the NaCl levels (Table 5). Titanium as an individual factor and the interactions between the factors had no significant effects on this variable. Importantly, in roots, treatments did affect this variable significantly (Table 6).

In leaves, the antioxidant activity was only reduced by 27% with the addition of 100 mM NaCl, compared to the control after 15 min. In stems, no significant effects were found at 15 and 30 min, and only the 100 mM NaCl dose increased the antioxidant activity by 50% compared to the control, after 60 min (Table 5).

In roots, the antioxidant activity increased with 50 mM NaCl at 15, 30, and 60 min by 39, 41, and 48%, respectively, compared to the control. On the contrary, the application of 100 mM NaCl reduced the antioxidant activity in all three measurements made, both with respect to the application of 50 mM NaCl and with respect to the control. Ti as an individual factor had no significant effect on this variable in any of the concentrations tested or times measured. In the interactions of the study factors, at 15 min, 30 and 60 min, the highest antioxidant activity was observed with the treatment containing 50 mM NaCl and 1000 mg Ti L^−1^ and the lowest activity with the treatment containing 100 mM NaCl and 1000 mg Ti L^−1^; in all cases, these treatments were statistically different from the control without salinity and without Ti (Table 6).

### 2.5. Nutrient Concentration

The main effect of NaCl on N concentration in tomato leaves was not significant. However, the concentration of P with the 100 mM NaCl dose was significantly higher than the control, by 21%. The K concentration was significantly lower than the control, by 28 and 32% with the 50 and 100 mM NaCl doses, respectively. Regarding the main effect of Ti, the concentrations of N, P, and K were significantly higher than the respective control. Specifically, N concentrations were 7 and 16% higher in plants exposed to 500 and 1000 mg Ti L^−1^, respectively, as compared to the control. P concentrations were 14.5 and 28% higher in plants exposed to 500 and 1000 mg Ti L^−1^, respectively, also as compared to the control. For K, its concentrations were 19 and 21% higher in plants exposed to 500 and 1000 mg Ti L^−1^, respectively, as compared to the control. The interaction of the study factors showed significant effects on N concentration. Plants exposed to 50 mM NaCl and 1000 mg Ti L^−1^ exhibited 28.5 and nearly 30% higher concentrations of N and P, respectively, as compared to the control. This combination also increased the Mg concentration by nearly 12% as compared to the control. On the other hand, the treatment with 100 mM NaCl and 1000 mg Ti L^−1^ significantly increased the concentrations of P and K, by 45 and 44%, respectively, when compared with the treatment with 100 mM NaCl and without Ti (Table 7).

Regarding the main effect of NaCl on Ca concentration in leaves, the application of 50 and 100 mM NaCl significantly decreased its concentration, by 7 and 16%, respectively, as compared to the control. The 100 mM NaCl dose significantly decreased the concentration of Mg and increased the concentration of Ti. As for the main effect of Ti on Ca concentration, the 500 mg Ti L^−1^ dose ensured the highest value with respect to the control. Both Ti doses (500 and 1000 mg Ti L^−1^) increased the concentration of Mg, by 14 and 11%, compared to the control. On the other hand, the Ti concentration increased along with the increase in leaf doses of TiO_2_ applied. The interaction of the study factors showed that the treatment with 100 mM NaCl and 1000 mg Ti L^−1^ reduced the Ca concentration by 18%, with respect to the treatment with 100 mM NaCl and without Ti. The treatment with 50 mM NaCl and 1000 mg Ti L^−1^ significantly increased the concentration of Mg, by 23%, with respect to the treatment with 50 mM NaCl without Ti. In the treatment with 100 mM NaCl and 1000 mg Ti L^−1^, a significant decrease in leaf Mg concentration (of nearly 40%) was observed, with respect to the treatment with 0 mM NaCl and 500 mg L^−1^ TiO_2_ that displayed the highest mean for this variable (Table 7).

## 3. Discussion

In plants, the growth process is negatively affected by excess salinity in the growth medium. Excess NaCl in the substrate increases osmotic pressure and reduces water absorption, decreasing cell turgor and elongation. Inside the plant, the Na^+^ cation and the Cl^−^ anion are concentrated in senescent leaves and through the flow of transpiration they are translocated to the aerial part, affecting the cells in leaves with greater photosynthetic activity and thus reducing the rate of photosynthesis, carbon uptake, and accumulation of fresh biomass [29]. In our study, salinity significantly affected the variables of seedling height, stem diameter, and leaf area (Table 1 and Table 2). Similarly, the application of 50 mM NaCl decreased plant height and stem diameter in hybrid Saladette-type “Pony Express” tomato seedlings [30], and 150 mg L^−1^ NaCl affected the leaf area of Chonto-type tomato seedlings [31]. This indicates that, at a higher NaCl concentration, lower seedling height, stem diameter, and leaf area are observed, that is, these variables are affected inversely proportional to the level of salinity tested [32,33,34].

The biological effects of Ti on plant physiology and metabolism (i.e., synthesis of the Ti chelating α-hydroxy carboxylic acids—citric and malic acids-; synthesis of ascorbic acid; modulation of Fe and Mg contents in plant tissues; chlorophyll biosynthesis; as well as enzymatic activity of nitrate reductase and of other proteins involved in the antioxidant system) are dose-dependent and therefore display hormesis [35,36]. Hormesis is a natural phenomenon characterized by favorable responses to low-level exposures to a chemical compound or to adverse conditions [37]. This phenomenon has a plethora of applications in different disciplines and can generally be utilized as a quantitative measure of biological plasticity through adaptive responses under stressful conditions [38]. In the primary producer, nitrogen-fixing cyanobacteria *Anabaena* PCC 7120, a nano-TiO_2_ dose-dependent production of amino acids involved in N assimilation and intracellular N storage was observed, which possibly contributes to the increase in newly synthesized proteins needed for detoxification in response to the cellular stresses induced by nano-TiO_2_ treatments in a hormetic manner [39]. Though approaches to address the uptake, translocation, phytotoxicity, and hormetic effects of nano-TiO_2_ have been undertaken [40], in-depth studies on the hormetic effects of bulk Ti on plant biology are lacking. Therefore, our study may contribute with novel data to generate the information needed to estimate the hermetic responses of plants to Ti applications.

In this research, the beneficial effect of Ti on the growth variables evaluated was not observed at the saline doses tested. Rather, it is likely that the doses of Ti applied to the tomato seedlings caused an additional stress to that already induced by salinity, which triggered negative effects on the variables evaluated (Table 1 and Table 2). Similar effects with Ti were reported in rice (*Oryza sativa* L.) and Narbon bean (*Vicia narbonensis*), since the exposure of plants to doses between 500 and 2000 mg Ti L^−1^ and 0.2% *n*TiO_2_ inhibited germinative and growth processes [41,42]. Potentially, Ti may induce toxicity in mitotic cell chromosomes [43]. Furthermore, it could negatively affect the activity of enzymes related to nitrogen metabolism (nitrate reductase, glutamate dehydrogenase, glutamine synthase, and glutamic-pyruvic transaminase), an essential element (i.e., the major plant macronutrient) highly related to growth and development in plants [44]. 

The accumulation of the Cl^−^ anion over time produces leaf necrosis, which decreases the photosynthetic capacity of the leaves and inhibits the absorption of nitrate. Similarly, excess Na^+^ exerts an antagonistic effect on K^+^, which regulates nitrate metabolism by acting as a companion ion and as an activator of the nitrate reductase enzyme, responsible for initiating the nitrate reduction process [45,46]. Similarly, by adding 50 mM NaCl to rough lemon (*Citrus jambhiri* Lush.), the mean value of SPAD units decreased [47]. Likewise, the addition of 20, 40, and 60 mM NaCl in common bean (*Phaseolus vulgaris* L.) cv. Ica Cerinza decreased SPAD units 28 days after the treatments were applied [48]. This negative effect of NaCl on SPAD units was evident in this study (Table 2).

Decreases in chlorophyll contents in seedlings under saline stress can be attributed to an increase in the activity of the enzyme chlorophyllase, in addition to the degradation of chloroplasts [49]. This was not observed in our study, since there were no significant differences in chlorophylls *a* or *b* among treatments (Table 3). Coincidentally, red radish (*Raphanus sativus* L.) seedlings at the V18 stage exposed to 70 mM NaCl did not decrease their chlorophyll concentrations, which was attributed to the sufficiency of N and Mg to cope with stress [50]. This result is also in full agreement with our study since there were no significant differences in N with the addition of NaCl (Table 7). Sodium chloride inhibits transpiration and stomatal conductance; it affects components of photosynthesis such as chlorophylls and carotenoids, and the quantum yield of PSII electron transport [51,52]. This effect was only observed in the total chlorophyll variable, since the treatment with 100 mM NaCl and 1000 mg Ti L^−1^ reduced the concentration of total chlorophyll with respect to the treatment without salinity with 500 mg Ti L^−1^ (Table 3).

Sugars are organic solutes associated with salt tolerance in higher plants. At the cellular level, an osmotic balance is created between the tonoplast and the external environment, through the synthesis and accumulation of sugars in the cytoplasm and vacuole, which allow tolerance to salinity through ionic homeostasis and water absorption [53,54,55,56]. On the other hand, salinity improves the accumulation and transport of carbohydrates within the plant, as it promotes the expression of the *LeSUT1* gene, which encodes a sucrose transporter from source leaves, through the phloem, to other organs such as stems and fruits [57,58]. In our study, the highest accumulation of sugars was observed in the treatments without salinity in leaves and roots. However, in stems, the concentration of sugars was higher in the treatment with 50 mM NaCl compared to that observed in seedlings exposed to the treatment without NaCl (Table 4). This finding coincides with a study carried out in tomato cv. INCA 17 treated with 50 mM NaCl, where increases in dry weight were observed, due to greater synthesis of sugars in the stems [59].

In this research, the leaf-applied TiO_2_ did not significantly affect the concentration of sugars in stems or roots, which is also in full agreement with what was observed in tomato fruits cv. ISI 68249 treated with 80, 240, 480, and 960 g Ti ha^−1^ [60]. Nevertheless, Ti did decrease the concentration of sugars in leaves (Table 4).

When a crop is challenged with biotic or abiotic stress factors, there is an overaccumulation of free radicals at the cellular level that cause cascade reactions, potentially damaging biomolecules such as proteins, lipids, and nucleic acids, which can lead to the activation of apoptotic processes. During the process of defense against oxidative damage, plants activate both enzymatic and non-enzymatic antioxidant systems. In tomato, the main antioxidant metabolites are lycopene and ascorbic acid, which are responsible for capturing most reactive oxygen species [61]. Table 5 and Table 6 show the evaluated antioxidant activity in leaves, stems, and roots. Of the three evaluated organs, the NaCl factor caused the greatest antioxidant activity in leaves, while the NaCl × Ti interaction increased this activity in roots exposed to 50 mM NaCl at every level of Ti tested. Among the possible antioxidant enzymes activated by the tested treatments, we can find superoxide dismutase (SOD), catalase (CAT) and various peroxidases (GPX and APX) [62,63]. In seedlings exposed to stress, Ti can contribute to reducing the concentration of reactive oxygen species through the potential stimulation of these enzymes [64]. 

In pepper, iron-depending enzymes as well as nitrate reductase were stimulated by Ti applications, thus activating the antioxidant system and protecting the plants against reactive oxygen species damage [65]. In different strawberry (*Fragaria* x *ananassa* Duch.) cultivars, the application of Ti increased the concentrations of L-ascorbic acid and total anthocyanins, while significant enhancements of scavenging 2,2’-azinobis-(3-ethylbenzothiazoline-6 sulfonic acid) (ABTS) and 2-diphenyl-1-picrylhydrazyl (DPPH) were also observed, demonstrating an increased antioxidant activity triggered by Ti in this species [66]. In henbane (*Hyoscyamus niger* L.), both nano-sized TiO_2_ (NT) and bulk TiO_2_ (BT) increased SOD activity with increasing TiO_2_ concentrations, whereas tropane alkaloid biosynthesis was also stimulated at low NT levels. In general, all tested antioxidant enzymes displayed higher activity in NT- treated plants, as compared to those of BT-treated ones [67]. In maize (*Zea mays* L.), Ti increased antioxidant enzyme activity and decreased malondialdehyde (MDA) accumulation, thus preventing lipid peroxidation and oxidative damage [68].

Saline increases in the growth media (i.e., soil, substrates or nutrient solutions) prevent plants from absorbing enough water, due to an increase in the osmotic pressure at the root level. In turn, the increased osmotic pressure provokes stomatal closure, decreased transpiration rate, or reduced nutrient load in the xylem [69]. Sodium chloride affects N absorption and metabolism, in addition to its transport and distribution to the aerial part, since the Cl^−^ anion has an antagonistic effect with both NO_3_^−^ and NH_4_^+^ [70,71,72]. However, this effect was not observed in our study, as N was not affected by the treatments with 50 and 100 mM NaCl. On the other hand, salinity enhanced the accumulation of P in leaves. This result is consistent with that observed in lavender (*Lavandula angustifolia* Mill.) exposed to 25 and 50 mM NaCl, which increased P concentrations in leaf tissues as compared to the control not exposed to NaCl [72]. Negative effects were observed in the leaf concentration of K, since salinity reduced it by 28 and 32%, with respect to the control. This is because the Na^+^ cation competes for binding sites in the transport of the K^+^ cation, in addition to the damage caused by Na^+^ in the cell membrane, allowing K^+^ leakage [73,74], as can be seen in Table 7.

The NaCl factor tended to reduce the concentrations of K, Ca, and Mg, but increased those of P and Ti. The Ti factor increased the concentrations of N, P, K, Mg, and Ti in comparison with each respective control. In the case of Ca, Ti increased its concentration only with 500 mg Ti L^−1^, in the absence of NaCl. The interaction of the study factors (NaCl × Ti) showed a higher mean of N when the seedlings were treated with 1000 mg Ti L^−1^, regardless of the NaCl value tested. The P concentration was higher when applying 100 mM NaCl and 1000 mg Ti L^−1^. The K concentration was higher with increasing Ti levels, in the absence of NaCl, which was also observed for Ca and Mg. The concentration of Ti was higher when applying 100 mM NaCl and 1000 mg Ti L^−1^ (Table 7). Similar effects have been reported in tomato cv. ISI 68249 treated with 960 g Ti ha^−1^ [60]. 

In some pioneering experiments with pepper, the concentration of some essential macro- and micronutrients were enhanced when plants were supplied with Ti [75]. Interestingly, Ti effects were maximal under field conditions with traditional fertilization and minimal in optimal conditions under controlled environments in hydroponics [75,76]. Coincidently, when withdrawing N or P from the fertilizer applied to Ti-treated crops, no nutritional imbalances in the plants were observed [77,78]. Importantly, the concentration of Fe in Ti-treated pepper plants was higher than in controls, and the increase was higher for Fe as compared to the other nutrients analyzed [79], which indicates that Fe may be a critical element implicated in the action mechanism of Ti in plants [80]. In peach (*Prunus persica* L.), Ti application showed significant increases in Fe, Cu and Zn concentrations in the peel, and Ca concentration in the peel and flesh [81,82], which can be explained by the beneficial effect of Ti on the processes of nutrient absorption, translocation and assimilation within the plant. In the M.26 EMLA apple (*Malus* sp.) rootstock, foliar Ti applications enhanced plant dry matter and levels of P, Fe, Mn, and Zn in leaf tissues, with leaves being greener and having higher Fe^2+^ and chlorophyll concentrations than those of control plants. These responses could be attributed to a higher concentration of Fe^2+^ in leaves promoted by Ti applications, which enhanced chlorophyll synthesis and uptake of essential nutrients [83]. In spinach (*Spinacia oleracea* L.), nano-TiO_2_ properly applied accelerated germination of naturally aged seeds and increased seedling vigor. Furthermore, nano-TiO_2_ improved formation of chlorophyll and enhanced the enzymatic activity of Rubisco and the photosynthetic rate as a result of improved uptake and absorption of essential nutrients such as N and Mg [84]. At typical Ti concentrations in the organism, Ti might exist as a labile pool of Ti(IV) within the cell, similar to Fe. Thus, Ti could mimic the functions of Fe in the cell. Various intracellular targets of Ti include phosphoproteins, DNA, ribonucleotide reductase, and ferritin [85]. We are currently further investigating the effects of Ti on Fe concentrations in different tissues of tomato and other horticultural crops to answer this question.

## 4. Materials and Methods

### 4.1. Plant Material, Saline and Leaf Treatments

Tomato (*Solanum lycopersicum* L.) seeds cv. Rio Grande were germinated in polyethylene containers with 200 cavities, using peatmoss as substrate. During this germination period, the moisture of the tray was maintained by sprinkling the seedlings with 500 mL of distilled water once a day. Thirty-one days after sowing (DAS), the obtained seedlings were transplanted into 250 cm^3^ Styrofoam cups previously filled with perlite. From the transplant, the seedlings were maintained with Steiner’s nutrient solution (NS) [86] at 25% of its original strength. At 28 days after transplantation (DaTr), the concentration of this solution was increased to 50%.

At 28 DaTr, saline treatments were started by applying 50 and 100 mM NaCl to the substrate through Steiner’s NS used in irrigation, in addition to a control, which consisted only of Steiner’s NS at 50%. At 29 DaTr, foliar applications of either 0, 500, or 1000 mg Ti L^−1^ (from TiO_2_.) were carried out. Irrigation was done manually using a beaker; 80 mL of NS per plant day^−1^ were applied during the first 28 DaTr. In the subsequent 15 days, 100 mL of NS plant day^−1^ were applied, and from there it was increased to 120 mL of NS plant day^−1^ for the remaining 21 days. Four foliar applications were carried out at 7-day intervals. Each application was done at 06:00 h, sprinkling the seedlings to the point of dripping. For the leaf sprays, Tween 20^®^ was used as a surfactant at a concentration of 0.5 g L^−1^.

### 4.2. Experimental Design

The experiment was conducted in a greenhouse with a top window. An experimental design with completely randomized distribution and a factorial treatment arrangement was used. The first factor was the NaCl concentration at three levels: 0, 50, and 100 mM, in the NS used for irrigation, while the second factor was the leaf applications of Ti at three concentrations: 0, 500, and 1000 mg Ti L^−1^, resulting in a total of nine treatments to be tested. Each treatment had three replicates. The experimental unit consisted of one plant established in a Styrofoam cup.

### 4.3. Growth Variables

Seedling height was measured 1, 10, 20, and 30 days after the start of the treatments. It was measured from the base of the stem to the growth apex with a tape measure (Truper 5 m Gripper; Shanghai, China). At the same time intervals, stem diameter was measured at 10 cm from the base using a digital Vernier caliper (Truper 14388; Shanghai, China). At 95 DAS, the seedlings were harvested. Leaf area was determined using a leaf area integrator (LI-CORLI-3000A; Lincoln, NE, USA). To obtain the weight of dry biomass, the seedlings were divided into roots, stems and leaves; later they were placed in a forced air oven (Riossa HCF-125D; Guadalajara, Jalisco, Mexico) at 70 °C. They were then weighed separately on an analytical scale (Adventurer Ohaus Pro AV213C; Parsippany, NJ, USA).

### 4.4. SPAD Units and Concentration of Photosynthetic Pigments

At 0, 10, 20, and 30 DAT, the SPAD (Soil Plant Analysis Development) units were measured with a portable SPAD-502^®^ meter (Minolta; Tokyo, Japan). To determine the concentrations of photosynthetic pigments (chlorophyll *a*, chlorophyll *b*, total chlorophyll and carotenoids), the methodology described by Sumanta et al. [87] was used. Fifty mg of plant material were taken and macerated with 10 mL of 95% (*v*/*v*) ethanol. The samples were placed in 15 mL Falcon tubes and centrifuged at 10,000 rpm in a refrigerated centrifuge (Eppendorf 5424; Eppendorf, Germany) for 5 min at 4 °C, and the supernatant was recovered in a new Falcon tube. From the supernatant obtained, 0.5 mL was taken and mixed with 4.5 mL of 95% ethanol. In these samples, the absorbance was measured in a spectrophotometer (Jenway 6715 UV/Vis; Staffordshire, UK) at 470, 649, and 664 nm. The concentrations of the photosynthetic pigments were determined with the following formulas:

C x + c = (1000A_470_ − 2.13Ca − 97.63C_b_)/209

Ch-a = 13.36A_664_ − 5.19A_649_

Ch-b = 27.43A_649_ − 8.12A_664_

The total chlorophyll concentration was the sum of chlorophylls *a* and *b*.

### 4.5. Determination of Total Sugars

At harvest (i.e., at 95 DAS), total soluble sugars were determined following the protocol described by Bayley [88]. Accordingly, 500 mg of the sample were weighed and 50 mL of 80% ethanol were added. The supernatant was obtained after shaking it in a heating plate (Thermo Scientific SP131635; Kuala Lumpur, Malaysia), filtered and brought to a final volume of 20 mL. The samples were incubated in a water bath at 95 °C for 15 min, and then the process was finished by placing the samples on ice. To quantify the total sugars, a standard curve was made using glucose (Sigma-Aldrich; Saint Louis, MO, USA), and they were measured at an absorbance of 600 nm in a spectrophotometer (Jenway 6715 UV/Vis).

### 4.6. Antioxidant Activity

At harvest (i.e., at 95 DAS), the methodology described by Ibarra et al. [89] was used to determine antioxidant activity. Accordingly, 100 mg of plant tissue, previously macerated and homogenized with liquid N, were weighed, 1.5 mL of 60% ethanol was added, and it was left to stand for 24 h in refrigeration at 3 °C. The following day, it was centrifuged at 15,000 rpm for 20 min at 4 °C; 400 µL of the supernatant and 600 µL of 80% methanol were placed in a reaction tube, and finally 1 mL of 2,2-diphenyl-1-picrylhydrazyl (DPPH) solution was added and stirred in a vortex. Samples were read in a Jenway 6715 UV/Vis spectrophotometer at a wavelength of 517 nm, 15, 30 and 60 min after the addition of DPPH. Trolox (6-hydroxy-2,5,7,8-tetramethylcoman-2-carboxylic acid) was used as a standard. Two blanks were used in the methodology, one to calibrate the spectrophotometer (400 µL of 60% ethanol plus 600 µL of 80% methanol) and the other to prepare the standard curve (without Trolox).

### 4.7. Nutrient Concentration

The protocols described by Alcántar and Sandoval [90] were used to carry out the nutritional analysis in plant tissues collected at harvest (i.e., at 95 DAS). Accordingly, 0.25 g of dry tissue were taken, to which a mixture of acids and hydrogen peroxide (H_2_SO_4_:HClO_4_:H_2_O_2_; 2:1;1, *v*:*v*) was added to carry out wet digestion. Once the digestion of the organic matter was complete, the sample was adjusted to 25 mL of deionized water and filtered. The concentration of N was determined by the micro-Kjeldahl method and the rest of the elements through readings with an inductively coupled plasma optical emission spectrophotometer (Varian ICP OES 725-ES; Mulgrave, Australia).

### 4.8. Statistical Analysis

With the results obtained, an analysis of variance was performed according to a factorial treatment design. Means comparisons were performed with Tukey’s test (*p* ≤ 0.05).

## 5. Conclusions

This study showed that salinity as an individual factor negatively affected the following variables: seedling height, stem diameter, leaf area, SPAD units, total sugars in leaves and roots, antioxidant activity, and the concentrations of K, Ca, and Mg in the tomato seedlings evaluated. Titanium increased the concentrations of N, P, K, Ca, Mg, and Ti, and stimulated higher antioxidant activity in roots. The interaction of the factors benefited the antioxidant activity in roots and increased the concentrations of N, P, and Ti in leaf tissues. These findings demonstrate a positive effect of titanium on the antioxidant activity and nutrition of tomato seedlings under saline stress conditions. As Ti triggers hormesis in plants, so a conditioning method utilizing it as a plant biostimulator is possibly a consistent, reasonable, and cost-effective means for enhancing plant growth and productivity in saline environments. Our findings provide a logical basis for suggesting that Ti contributes to the alleviation of salt stress in tomato. Additional studies are necessary to determine the precise functions of Ti under salinity. 

## Figures and Tables

**Table 1:** Main and interaction effects of NaCl and TiO_2_ on seedling height and stem diameter of tomato (*Solanum lycopersicum* L.) cv. Rio Grande.

Source of Variation		Seedling Height (cm)			Stem Diameter (mm)		
		10 DAT	20 DAT	30 DAT	10 DAT	20 DAT	30 DAT
**NaCl (mM)**							
**0**		36.2 ± 1.4 a	46.4 ± 5.4 a	59.1 ± 3.6 a	6.0 ± 0.3 a	6.5 ± 0.2 a	6.9 ± 0.3 a
**50**		32.5 ± 1.9 b	37.4 ± 1.5 b	39.7 ± 2.1 b	5.5 ± 0.2 b	5.7 ± 0.2 b	5.9 ± 0.2 b
**100**		27.9 ± 1.3 c	31.6 ± 1.2 c	32.9 ± 1.2 c	5.2 ± 0.2 b	5.4 ± 0.1 b	5.8 ± 0.2 b
**Ti (mg L^−1^)**							
**0**		32.2 ± 2.1 a	39.7 ± 3.8 a	45.1 ± 6.8 a	5.6 ± 0.2 a	5.9 ± 0.2 a	6.2 ± 0.3 a
**500**		33.0 ± 2.6 a	37.6 ± 5.5 a	44.3 ± 5.0 a	5.6 ± 0.3 a	5.9 ± 0.4 a	6.2 ± 0.3 a
**1000**		31.3 ± 2.2 a	38.1 ± 3.9 a	44.3 ± 5.1 a	5.5 ± 0.2 a	5.7 ± 0.3 a	6.1 ± 0.4 a
**NaCl (mM)**	**Ti (mg L^−1^)**						
**0**	**0**	35.8 ± 1.1 a	48.9 ± 1.1 a	60.6 ± 5.7 a	5.8 ± 0.2 ab	6.2 ± 0.2 abc	6.6 ± 0.4 abc
**0**	**500**	36.9 ± 2.1 a	41.9 ± 9.3 abc	57.0 ± 1.9 a	6.4 ± 0.2 a	6.8 ± 0.3 a	7.0 ± 0.1 a
**0**	**1000**	35.8 ± 0.7 a	48.3 ± 1.2 ab	59.7 ± 2.7 a	5.7 ± 0.2 ab	6.4 ± 0.2 ab	7.0 ± 0.3 ab
**50**	**0**	31.3 ± 2.4 abc	37.6 ± 2.3 abc	41.2 ± 2.9 b	5.6 ± 0.2 ab	5.9 ± 0.2 bcd	6.2 ± 0.3 abc
**50**	**500**	34.3 ± 2.2 ab	38.9 ± 0.8 abc	41.6 ± 0.8 b	5.2 ± 0.1 b	5.5 ± 0.1 dc	5.8 ± 0.2 c
**50**	**1000**	31.9 ± 0.6 abc	35.9 ± 0.8 bc	36.2 ± 1.0 bc	5.7 ± 0.3 ab	5.7 ± 0.2 bcd	5.7 ± 0.2 c
**100**	**0**	29.5 ± 1.6 bc	32.6 ± 1.6 c	33.6 ± 1.1 bc	5.5 ± 0.2 ab	5.6 ± 0.1 bcd	5.9 ± 0.1 bc
**100**	**500**	27.9 ± 1.0 c	32.1 ± 0.9 c	34.4 ± 1.3 bc	5.1 ± 0.2 b	5.4 ± 0.2 cd	5.7 ± 0.3 c
**100**	**1000**	26.1 ± 1.0 c	30.2 ± 0.5 c	30.9 ± 0.5 c	5.0 ± 0.1 b	5.2 ± 0.1 d	5.7 ± 0.2 c

DAT = days after treatments; Means ± SD with different letters in each column and study factor indicate statistically significant differences between treatments (Tukey, *p* ≤ 0.05).

**Table 2:** Main and interaction effects of NaCl and TiO_2_ on leaf area and SPAD units of tomato (*Solanum lycopersicum* L.) seedlings cv. Rio Grande.

Source of Variation		Leaf Area (cm^2^)	SPAD Units		
			10 DAT	20 DAT	30 DAT
**NaCl (mM)**					
0		518.7 ± 20 a	49.3 ± 1.8 ab	54.4 ± 2.0 a	52.8 ± 2.9 a
50		276.2 ± 46 b	50.4 ± 2.6 ab	50.5 ± 3.6 ab	45.8 ± 2.8 b
100		157.8 ± 25 c	46.3 ± 2.3 b	47.8 ± 3.2 b	34.7 ± 5.4 c
**Ti (mg L^−1^)**					
0		340.6 ± 76 a	49.3 ± 2.8 a	49.8 ± 3.1 a	43.1 ± 4.1 a
500		322.7 ± 90 a	48.1 ± 2.0 a	50.0 ± 2.8 a	43.7 ± 5.1 a
1000		289.5 ± 88 a	48.5 ± 2.4 a	52.9 ± 3.7 a	46.5 ± 6.8 a
**NaCl (mM)**	**Ti (mg L^−1^)**				
0	0	503.8 ± 21 ab	47.9 ± 2.2 a	51.3 ± 2.0 a	49.2 ± 4.5 ab
0	500	547.4 ± 19 a	49.9 ± 1.6 a	54.4 ± 1.8 a	53.4 ± 1.6 a
0	1000	504.9 ± 17 ab	50.0 ± 1.6 a	57.5 ± 0.7 a	55.9 ± 0.5 a
50	0	340.5 ± 51 bc	52.3 ± 3.3 a	50.5 ± 3.6 a	41.9 ± 2.9 abc
50	500	244.0 ± 48 cd	48.2 ± 2.4 a	49.6 ± 2.9 a	44.9 ± 2.0 abc
50	1000	244.2 ± 34 cd	50.7 ± 2.0 a	51.6 ± 4.7 a	50.6 ± 1.8 ab
100	0	177.4 ± 19 cd	47.8 ± 2.7 a	47.6 ± 3.6 a	38.2 ± 3.0 bc
100	500	176.6 ± 27 cd	46.2 ± 1.7 a	46.0 ± 2.3 a	32.8 ± 4.3 c
100	1000	119.2 ± 25 c	44.7 ± 2.5 a	49.8 ± 3.9 a	33.1 ± 8.2 c

DAT = days after treatments; Means ± SD with different letters in each column and study factor indicate statistically significant differences between treatments (Tukey, *p* ≤ 0.05).

**Table 3:** Main and interaction effects of NaCl and TiO_2_ on photosynthetic pigments in leaves of tomato (*Solanum lycopersicum* L.) seedlings cv. Rio Grande.

Source of Variation		Chlorophyll *a*	Chlorophyll *b*	Total Chlorophyll	Carotenoids
		(µg g^−1^ FW)			
**NaCl (mM)**					
0		36.8 ± 5 a	15.9 ± 1 a	52.8 ± 3.1 a	9.0 ± 1.3 a
50		36.3 ± 4 a	15.7 ± 1 a	53.4 ± 2.3 a	9.2 ± 0.8 a
100		31.9 ± 2 a	12.1 ± 3 a	44.1 ± 2.1 a	7.7 ± 1.2 a
**Ti (mg L^−1^)**					
0		34.1 ± 3 a	15.6 ± 0.6 a	49.7 ± 3.2 a	8.2 ± 0.9 a
500		38.4 ± 5 a	15.6 ± 3.1 a	54.1 ± 3.5 a	9.2 ± 1.5 a
1000		33.9 ± 3 a	12.5 ± 2.0 a	46.4 ± 4.0 a	8.6 ± 0.8 a
**NaCl (mM)**	**Ti (mg L^−1^)**				
0	0	32.4 ± 5 a	14.8 ± 0.5 a	47.3 ± 5.4 ab	8.0 ± 1.7 a
0	500	47.7 ± 2 a	19.0 ± 0.3 a	66.7 ± 2.1 a	11.4 ± 0.4 a
0	1000	30.4 ± 1 a	13.8 ± 1.1 a	44.3 ± 2.6 ab	7.6 ± 0.7 a
50	0	36.1 ± 2 a	16.1 ± 0.7 a	52.3 ± 2.1 ab	8.4 ± 0.8 a
50	500	36.4 ± 6 a	14.9 ± 1.5 a	51.3 ± 3.5 ab	9.3 ± 1.0 a
50	1000	40.3 ± 2 a	16.0 ± 1.1 a	56.4 ± 4.9 ab	10.0 ± 0.7 a
100	0	33.7 ± 1 a	15.7 ± 0.7 a	49.4 ± 1.9 ab	8.1 ± 0.2 a
100	500	31.1 ± 3 a	13.0 ± 2.5 a	44.2 ± 3.5 ab	6.9 ± 2.2 a
100	1000	30.9 ± 2 a	7.7 ± 2.0 a	38.6 ± 0.9 b	8.1 ± 0.8 a

FW = fresh weight. Means ± SD with different letters in each column and study factor indicate statistically significant differences between treatments (Tukey, *p* ≤ 0.05).

**Table 4:** Main and interaction effects of NaCl and TiO_2_ on sugar concentration on tomato (*Solanum lycopersicum* L.) seedlings cv. Rio Grande.

Source of Variation		Leaves	Stems	Roots
		(g 100 g^−1^ FW)		
**NaCl (mM)**				
0		1.06 ± 0.10 a	1.87 ± 0.2 b	0.52 ± 0.06 a
50		0.78 ± 0.08 b	2.43 ± 0.3 a	0.43 ± 0.03 ab
100		1.01 ± 0.11 a	1.61 ± 0.1 b	0.36 ± 0.07 b
**Ti (mg L^−1^)**				
0		1.01 ± 0.07 a	2.43 ± 0.3 a	0.48 ± 0.06 a
500		0.75 ± 0.07 b	1.83 ± 0.2 a	0.40 ± 0.04 a
1000		1.08 ± 0.12 a	1.73 ± 0.2 a	0.44 ± 0.08 a
**NaCl (mM)**	**Ti (mg L^−1^)**			
0	0	1.12 ± 0.07 ab	1.90 ± 0.20 bc	0.54 ± 0.07 a
0	500	0.83 ± 0.06 bc	1.41 ± 0.03 cd	0.43 ± 0.04 a
0	1000	1.23 ± 0.06 a	2.29 ± 0.12 b	0.58 ± 0.07 a
50	0	0.90 ± 0.08 abc	3.32 ± 0.03 a	0.39 ± 0.04 a
50	500	0.66 ± 0.07 c	2.19 ± 0.21 b	0.44 ± 0.04 a
50	1000	0.77 ± 0.05 bc	1.77 ± 0.15 bcd	0.48 ± 0.03 a
100	0	1.01 ± 0.03 abc	1.82 ± 0.09 bcd	0.51 ± 0.07 a
100	500	0.77 ± 0.06 bc	1.87 ± 0.10 bcd	0.32 ± 0.03 a
100	1000	1.24 ± 0.06 a	1.14 ± 0.06 d	0.27 ± 0.04 a

FW = fresh weight. Means ± SD with different letters in each column and study factor indicate statistically significant differences between treatments (Tukey, *p* ≤ 0.05).

**Table 5:** Main and interaction effects of NaCl and TiO_2_ on antioxidant activity at 15, 30, and 60 min in leaves and stems of tomato (*Solanum lycopersicum* L.) seedlings cv. Rio Grande.

Source of Variation		15 min	30 min	60 min	15 min	30 min	60 min
		(mg g^−1^ FW)			(mg g^−1^ FW)		
**NaCl (mM)**			**Leaves**			**Stems**	
0		0.70 ± 0.09a	0.73 ± 0.08 a	0.75 ± 0.07 a	0.11 ± 0.04 a	0.17 ± 0.05 a	0.26 ± 0.06 b
50		0.63 ± 0.12 ab	0.66 ± 0.10 a	0.69 ± 0.08 a	0.28 ± 0.09 a	0.38 ± 0.11 a	0.48 ± 0.10 ab
100		0.51 ± 0.21b	0.57 ± 0.21 a	0.62 ± 0.20 a	0.32 ± 0.11 a	0.41 ± 0.10 a	0.52 ± 0.09 a
**Ti (mg L^−1^)**							
0		0.67 ± 0.11a	0.71 ± 0.10 a	0.73 ± 0.08 a	0.18 ± 0.07 a	0.27 ± 0.08 a	0.40 ± 0.09 a
500		0.64 ± 0.15a	0.69 ± 0.13 a	0.72 ± 0.10 a	0.21 ± 0.08 a	0.29 ± 0.09 a	0.40 ± 0.05 a
1000		0.54 ± 0.0.20 a	0.58 ± 0.19 a	0.61 ± 0.19 a	0.32 ± 0.07 a	0.41 ± 0.08 a	0.47 ± 0.06 a
**NaCl (mM)**	**Ti (mg L^−1^)**						
0	0	0.71 ± 0.02a	0.75 ± 0.01 a	0.77 ± 0.01 a	0.10 ± 0.05 a	0.15 ± 0.07 a	0.15 ± 0.09 a
0	500	0.75 ± 0.01a	0.77 ± 0.01 a	0.78 ± 0.01 a	0.05 ± 0.02 a	0.13 ± 0.01 a	0.13 ± 0.01 a
0	1000	0.64 ± 0.07a	0.68 ± 0.07 a	0.70 ± 0.06 a	0.17 ± 0.03 a	0.25 ± 0.04 a	0.25 ± 0.05 a
50	0	0.68 ± 0.04a	0.68 ± 0.03 a	0.74 ± 0.02 a	0.26 ± 0.09 a	0.38 ± 0.08 a	0.38 ± 0.09 a
50	500	0.61 ± 0.07a	0.68 ± 0.06 a	0.70 ± 0.05 a	0.27 ± 0.08 a	0.30 ± 0.07 a	0.30 ± 0.11 a
50	1000	0.60 ± 0.07a	0.68 ± 0.06 a	0.65 ± 0.05 a	0.32 ± 0.08 a	0.47 ± 0.10 a	0.47 ± 0.09 a
100	0	0.62 ± 0.10a	0.68 ± 0.09 a	0.69 ± 0.07 a	0.17 ± 0.05 a	0.29 ± 0.04 a	0.29 ± 0.05 a
100	500	0.54 ± 0.09 a	0.68 ± 0.09 a	0.68 ± 0.07 a	0.31 ± 0.07 a	0.43 ± 0.06 a	0.43 ± 0.06 a
100	1000	0.38 ± 0.10a	0.68 ± 0.08 a	0.48 ± 0.11 a	0.48 ± 0.16 a	0.51 ± 0.17 a	0.51 ± 0.15 a

FW= fresh weight. Means ± SD with different letters in each column indicate statistically significant differences between treatments (Tukey, *p* ≤ 0.05).

**Table 6:** Main and interaction effects of NaCl and TiO_2_ on antioxidant activity at 15, 30, and 60 min in roots of tomato (*Solanum lycopersicum* L.) seedlings cv. Rio Grande.

Source of Variation		15 min	30 min	60 min
		(mg g^−1^ FW)		
**NaCl (mM)**				
0		0.31 ± 0.02 b	0.29 ± 0.02 b	0.27 ± 0.02 b
50		0.43 ± 0.03 a	0.41 ± 0.02 a	0.40 ± 0.02 a
100		0.11 ± 0.01 c	0.11 ± 0.01 c	0.12 ± 0.01 c
**Ti( mg L^−1^)**				
0		0.27 ± 0.06 a	0.27 ± 0.06 a	0.26 ± 0.05 a
500		0.28 ± 0.06 a	0.27 ± 0.06 a	0.26 ± 0.05 a
1000		0.29 ± 0.08 a	0.28 ± 0.08 a	0.27 ± 0.07 a
**NaCl (mM)**	**Ti (mg L^−1^)**			
0	0	0.27 ± 0.03 c	0.25 ± 0.03 cd	0.23 ± 0.03 cd
0	500	0.33 ± 0.01 bc	0.31 ± 0.01 bc	0.29 ± 0.01 bc
0	1000	0.32 ± 0.01 bc	0.31 ± 0.01 bc	0.30 ± 0.01 bc
50	0	0.42 ± 0.04 ab	0.40 ± 0.04 ab	0.39 ± 0.04 ab
50	500	0.40 ± 0.03 abc	0.39 ± 0.02 ab	0.37 ± 0.02 ab
50	1000	0.47 ± 0.01 a	0.45 ± 0.01 a	0.44 ± 0.01 a
100	0	0.13 ± 0.01 d	0.14 ± 0.01 de	0.15 ± 0.01 de
100	500	0.11 ± 0.01 d	0.11 ± 0.01 e	0.12 ± 0.01 de
100	1000	0.08 ± 0.01 d	0.08 ± 0.02 e	0.09 ± 0.02 e

FW = fresh weight. Means ± SD with different letters in each column and study factor indicate statistically significant differences between treatments (Tukey, *p* ≤ 0.05).

**Table 7:** Main and interaction effects of NaCl and TiO_2_ on the concentration of N, P, K, Ca, Mg, and Ti in leaves of tomato (*Solanum lycopersicum* L.) seedlings cv. Rio Grande.

Source of Variation		N	P	K	Ca	Mg	Ti
		(g kg^−1^ DW)					(mg kg^−1^ DW)
**NaCl (mM)**							
0		22.7 ± 1.7 a	1.88 ± 0.10 b	11.9 ± 0.9 a	20.6 ± 1.5 a	4.82 ± 0.4 a	792 ± 77.3 b
50		22.5 ± 0.7 a	1.99 ± 0.06 b	8.6 ± 0.4 b	19.1 ± 0.3 b	4.80 ± 0.2 a	895 ± 97.8 b
100		22.1 ± 0.6 a	2.28 ± 0.19 a	8.1 ± 0.8 b	17.3 ± 0.8 c	3.86 ± 0.2 b	1445 ± 156.0 a
**Ti (mg L^−1^)**							
0		20.6 ± 0.9 c	1.79 ± 0.06 c	8.4 ± 0.60 b	18.4 ± 0.6 b	4.14 ± 0.1 b	19 ± 1.7 c
500		22.1 ± 0.3 b	2.05 ± 0.06 b	10.0 ± 1.30 a	19.9 ± 1.6 a	4.73 ± 0.3 a	1072 ± 144 b
1000		24.6 ± 0.8 a	2.30 ± 0.17 a	10.2 ± 1.20 a	18.6 ± 1.1 b	4.61 ± 0.4 a	2042 ± 336 a
**NaCl (mM)**	**Ti (mg L^−1^)**						
0	0	19.0 ± 1.2 c	1.62 ± 0.01 d	9.5 ± 0.05 b	17.0 ± 0.04 cd	3.73 ± 0.05 de	16 ± 0.7 e
0	500	22.8 ± 0.4 b	1.97 ± 0.06 bc	13.4 ± 0.11 a	23.8 ± 0.80 a	5.57 ± 0.23 a	941 ± 30 d
0	1000	26.3 ± 0.2 a	2.06 ± 0.02 bc	13.0 ± 0.26 a	20.9 ± 0.09 b	5.18 ± 0.03 ab	1419 ± 3 c
50	0	21.7 ± 0.3 bc	1.84 ± 0.02 cd	8.9 ± 0.25 bc	19.0 ± 0.10 bc	4.35 ± 0.17 cd	19 ± 1 e
50	500	22.1 ± 0.2 bc	2.01 ± 0.03 bc	9.5 ± 0.13 b	18.9 ± 0.34 bc	4.70 ± 0.07 bc	848 ± 28 d
50	1000	24.4 ± 0.1 ab	2.10 ± 0.02 bc	7.5 ± 0.18 cd	19.2 ± 0.53 bc	5.34 ± 0.07 ab	1820 ± 49 b
100	0	21.7 ± 0.4 bc	1.90 ± 0.04 bcd	7.0 ± 0.47 d	19.3 ± 0.67 bc	4.33 ± 0.08 cd	23 ± 0.7 e
100	500	21.6 ± 0.1 bc	2.18 ± 0.03 b	7.3 ± 0.27 d	16.8 ± 0.22 cd	3.93 ± 0.09 de	1426 ± 98 c
100	1000	23.1 ± 0.9 ab	2.75 ± 0.12 a	10.1 ± 0.16 b	15.8 ± 0.09 d	3.30 ± 0.02 e	2887 ± 138 a

DW = dry weight. Means ± SD with different letters in each column and study factor indicate statistically significant differences between treatments (Tukey, *p* ≤ 0.05).

## Data Availability

Not applicable.
